# Differential CpG methylation in the *Interferon Gamma* (*IFNG*) promoter correlates with viral load and transcriptional control in people with HIV

**DOI:** 10.1080/15592294.2026.2693337

**Published:** 2026-06-30

**Authors:** Megha S. Srivatsa, Momtahina Tahmida, Tara Tenenbaum, Susan J. Little, Brian LaMere, Sara Gianella, Sarah A. LaMere

**Affiliations:** a Department of Medicine, Division of Infectious Diseases and Global Public Health, University of California San Diego, San Diego, CA, USA; b Independent Scholar, Escondido, CA, USA

**Keywords:** DNA methylation, HIV, interferon gamma

## Abstract

HIV persistence is facilitated by immune evasion strategies that reshape host immune responses. As the *Interferon Gamma* (*IFNG*) gene encodes a key immunoregulatory cytokine whose transcriptional competence is governed by promoter CpG methylation, we hypothesized that HIV plasma RNA would track with epigenetic remodeling at the *IFNG* promoter, altering IFNG mRNA transcription. DNA methylation at the *IFNG* promoter was quantified by bisulfite sequencing in PBMCs from people with HIV (PWH) with high (*n* = 16; >5000 copies/µL), medium (*n* = 18; 500 to 4999 copies/µL), or undetectable (*n* = 18; ≤50 copies/µL) plasma HIV RNA. Corresponding IFNG mRNA was quantified by RT-qPCR, with a sensitivity analysis adjusting for T cell abundance. High detectable plasma HIV RNA was associated with significant *IFNG* hypomethylation across 4 out of 5 CpG sites in the *IFNG* promoter compared to those with medium HIV RNA, while those with undetectable plasma HIV RNA showed no significant differences after adjusting for duration of infection. Accordingly, IFNG mRNA transcription was significantly elevated in participants with high plasma HIV RNA compared to medium (*p* = 0.037), which strengthened after modeling TCF7 expression as a covariate to account for varying T cell counts (*p* = 0.02). These findings provide the first *in vivo* evidence correlating HIV viral load with epigenetic transcriptional control of *IFNG* in peripheral blood.

## Introduction

Despite effective antiretroviral therapy (ART), HIV persists through mechanisms that dampen or redirect host immune responses [1–3], suggesting that HIV leverages immunoregulatory strategies to maintain its reservoir. However, the molecular mechanisms that enable immunoregulation remain poorly understood. Epigenetic remodeling represents one such strategy, as chromatin modifications, such as DNA methylation [4–6], are known to shape both proviral latency [5,7–9] and host gene expression [10,11].

Prior studies have demonstrated HIV-associated chromatin modifications across immunoregulatory loci, particularly within genes involved within IFN signaling [12], but their *in vivo* consequences remain incompletely defined. Interferon-γ (IFN-γ) is a key early-response antiviral cytokine produced by Natural Killer (NK) cells, CD8+ cytotoxic T lymphocytes, and Th1 CD4+ T cells during HIV infection. Although IFN-γ does not directly inhibit HIV replication, it plays a central role in immune-mediated viral control through macrophage activation, antigen presentation, and T-cell differentiation [13]. *IFNG* is also among the most epigenetically regulated cytokine genes in human immunity [14–16], with promoter CpG methylation tightly linked to transcriptional competence across lymphocyte subsets [17]. These regulatory features make *IFNG* promoter methylation a sensitive epigenetic readout of immune activation states *in vivo*.

Clinical studies attempting to characterize how HIV infection perturbs IFN-γ expression have yielded mixed results. For example, acute HIV infection is marked by elevated IFN-γ in plasma [18–21], which decreases after the suppression of HIV RNA via ART initiation [18,19]. In contrast, PBMCs from viremic individuals produce less IFN-γ *ex vivo* [22–24]. Subset analysis shows that NK cells are the most efficient IFN-γ producers, followed by CD8^+^ T cells [25,26], with some evidence of increased IFNG mRNA transcription in CD4^+^ T cells [27]. These observations highlight cell-specific variability in cellular IFN-γ responses during HIV infection, but the underlying molecular mechanisms are yet to be established.

To elucidate potential mechanisms regulating IFN-γ expression during viral infection, we hypothesized that HIV antigen exposure influences *IFNG* promoter methylation *in vivo*, thereby modulating IFNG transcription. To test this, we quantified CpG methylation in the 5 CpGs in the *IFNG* promoter and IFNG mRNA in PBMCs from people with HIV across a spectrum of viral loads. To our knowledge, these findings provide the first *in vivo* evidence that plasma HIV RNA correlates with *IFNG* promoter hypomethylation and elevated IFNG transcription, suggesting that a viral load-responsive epigenetic mechanism shapes host immune responses.

## Materials and methods

### Ethics statement

Study samples were obtained retrospectively from participants enrolled in the San Diego Primary Infection Resource Consortium (PIRC), who provided written informed consent for the use of biological samples and clinical data. The study was approved by the Institutional Review Board at the University of California San Diego (IRB protocol #191088). All procedures were conducted in accordance with the Declaration of Helsinki.

### Participant samples

Study participants (*n* = 52) were enrolled in the San Diego Primary Infection Resource Consortium (PIRC) between 1996 and 2019. Participants were selected based on high (*n* = 16; >5000 copies), medium (*n* = 18; 500 to 4999 copies), and undetectable (*n* = 18; ≤50 copies) HIV RNA in plasma (Table 1 , Supplementary Table S1). All participants with undetectable HIV RNA were on suppressive ART, while the majority of participants with medium and high HIV RNA were not on ART (Table 1).

**Table 1.** Participant characteristics representing age, sex, race, ethnicity, risk factor for HIV acquisition, viral load, and CD4 T cell count at the time of sample draw.

	High VL	Medium VL	Undetectable VL
Age (at draw)	30 (27,28)	35 (29, 30)	41 (31, 46)
Sex			
Male	16	17	16
Female	0	1	2
Race			
White	11	16	15
Black	2	0	1
Other	3	2	2
Ethnicity			
Hispanic	2	2	6
Non-hispanic	14	16	12
Risk Factor			
MSM	14	17	16
Heterosexual/Other	2	1	2
Injection Drug User	2	1	0
ART Status			
On ART at Draw	2	5	18
Not on ART at Draw	14	13	0
Duration of Infection (Days)	27.5 (0,175)	72 (32.75, 487.5)	870.5 (339, 1827.75)
Viral Load	145,500 (49247, 262,163)	2497 (1551, 3979)	<50
CD4 Count	484 (387, 715)	555 (497, 726)	776 (497, 1054)

### Bisulfite treatment, Illumina library preparation, and sequencing

Genomic DNA and RNA were extracted from PBMCs using the Qiagen AllPrep DNA/RNA Mini Kit. DNA was spiked with unmethylated lambda control and subjected to bisulfite treatment with the Zymo Gold EZ DNA Methylation Kit. Primers targeting the *IFNG* promoter 557 bp upstream from the *IFNG* transcriptional start site (forward primer: 5’ – ATT GGG TAG TGT TGA TTT AGA GTA ATT TG − 3;’ reverse primer: 5’ – CTA CAC CTC CTC TAA CTA CTA ATA TTT ATA CC − 3’), and the lambda control target (forward primer: 5’ – TTG TGA TTA TTT TGG GTG AGG G − 3;’ reverse primer: 5’ – CAA TA TCT TCT TTA CAT TCA CCA CAC C − 3’), were designed using Primer3, and included adapter sequences compatible with Illumina sequencing applications.

Bisulfite PCR products were barcoded using i5 and i7 primers from the Illumina Nextera XT Index Kit v2, and amplified the KAPA HiFi Hotstart PCR Kit. Sequencing libraries were quantified using the Tapestation 2200 and Qubit systems. Amplicons were sequenced on the NextSeq 2000 to a depth of >1 million reads per sample. All lambda controls showed >98 % conversion and no difference between groups.

### Gene expression analysis

Relative RNA expression was determined using a SYBR Green quantitative reverse transcriptase PCR (RT-qPCR) for IFNG mRNA (forward primer: 5’ – CAA TAG CAA AAA GAA ACG AGA TGA C − 3;’ reverse primer: 5’ – GCG ACA GTT CAG CCA TCA CTT G − 3’), normalized to ribonuclease subunit P30 (RPP30) as a stable reference gene (forward primer: 5’ – GCG GAG GGA AGC TCA TCA G − 3;’ reverse primer: 5’ – GGA CAT GGG AGT GGA GTG ACA − 3’), and to T-Cell-Factor 7 (TCF7) to account for varying T cell counts among participants (forward primer: 5’ – CGA GGG AAA AGC ACC AAG AAT C − 3;’ reverse primer: 5’ – GCA CTG TCA TCG GAA GGA ACG − 3’).

### Bioinformatic analysis and statistics

For differential DNA methylation analysis, FASTQ files were preprocessed using Trim Galore! [28] for adapter trimming and aligned to the *IFNG* promoter using bwa-meth [29]. CpG methylation counts were extracted using MethylDackel [30]. Percent methylation at each CpG site was calculated from bisulfite sequencing as the proportion of methylated reads among total reads at that site. Differential methylation was modeled using a beta-binomial regression framework to account for overdispersion in methylation proportions across samples [31]. Specifically, methylated and unmethylated read counts were modeled using a beta-binomial generalized linear model (glmmTMB) with viral load group, age, duration of infection, CpG position, and group × CpG interaction terms included as fixed effects. CpG sites were treated as categorical variables to permit site-specific estimation of group effects. Post hoc pairwise contrasts between viral load groups were performed within each CpG site with multiple-comparison correction and estimated marginal means with Tukey-adjusted *p*-values. Percent methylation values reported in figures and tables represent descriptive means, while statistical significance was assessed using model-based contrasts.

Gene transcripts were quantified using ΔΔCt normalization. Following normalization to RPP30 expression, IFNG expression from medium and high viral load participants was calculated relative to the undetectable group. Statistical significance in expression differences were initially analyzed with Kruskal – Wallis and Dunn’s post-hoc tests with an alpha of 0.05, using GraphPad Prism 10.4.2 for MacOS [32]. To further assess whether observed IFNG expression differences were independent of variation in T cell abundance, linear regression models were performed incorporating TCF7 expression as a covariate. For these analyses, IFNG and TCF7 expression values normalized to RPP30 were analyzed using ΔCt values. Pairwise group comparisons were estimated using marginal means with Tukey-adjusted *p*-values.

Group differences in demographic and clinical covariates were assessed using Kruskal-Wallis tests with Dunn’s post hoc correction for continuous variables and Fisher’s exact tests for categorical variables. As viral suppression in this cohort was achieved through ART and ART status was highly colinear with viral load group, ART was not included as an independent covariate in primary methylation models. To further address potential ART-associated confounding, sensitivity analyses examining correlations between plasma HIV RNA and *IFNG* promoter methylation were restricted to participants not receiving ART.

Correlation analyses between plasma HIV RNA, *IFNG* promoter methylation, and IFNG transcription were performed using Spearman’s rank correlation. Viral plasma RNA values were log_10_ transformed for continuous analyses. Partial Spearman correlation analysis adjusted for duration since infection was additionally performed for participants not receiving ART.

## Results

### Cohort characteristics

The participant cohort was comprised primarily of cisgender men (*n* = 49) and included a subset of cisgender women (*n* = 3) with a median age of 35 (28, 33). Duration since HIV diagnosis differed significantly across viral load groups, with participants in the undetectable viral load group having longer durations of infection (Table 1, Supplementary Tables S1 and S2). Age did not differ significantly between groups. Risk factors for HIV acquisition primarily included men who have sex with men (MSM) (*n* = 47), heterosexual intercourse (*n* = 5) or intravenous drug use (*n* = 3). Racial and ethnic demographics of the cohort included Non-Hispanic Caucasian (*n* = 30), Black (*n* = 3), other racial minorities (*n* = 7), and Hispanic (*n* = 10) participants (Table 1). Participants were grouped by high (*n* = 16; median 145,500 HIV RNA copies/µl) medium (*n* = 18; median 2497 HIV RNA copies/µl), and undetectable (*n* = 18; <50 HIV RNA copies/µl) plasma HIV RNA expression. Participants with undetectable viral loads were all on suppressive ART, while the 27 of the 34 participants with detectable (medium and high) plasma HIV RNA were not on ART (Table 1 , Supplementary Table S1).

### Differential CpG methylation corresponds to viral load

**Figure 1.**
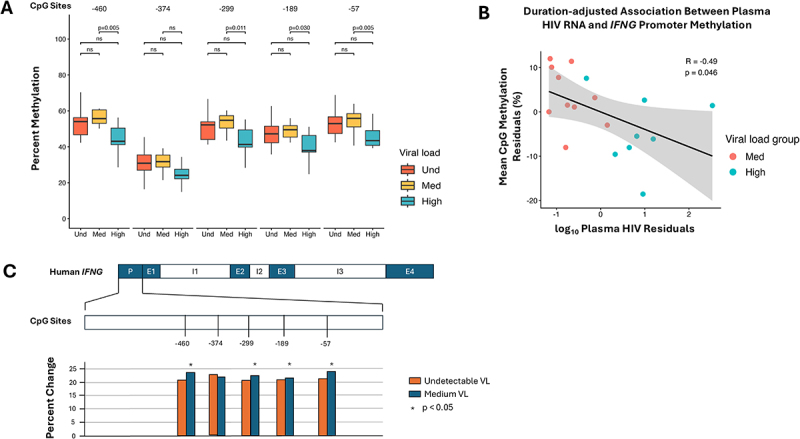
CpG methylation within the human *IFNG* promoter inversely correlates with plasma HIV RNA in PWH. (A) Absolute percent methylation at individual *IFNG* promoter CpG sites across viral load groups. Significant differences in methylation between viral load groups were identified using beta-binomial models adjusted for age and duration since infection. (B) Among participants not receiving ART, duration-adjusted partial Spearman correlation analysis demonstrated an inverse association between plasma HIV RNA and mean *IFNG* promoter methylation (*R* =  −0.49, *p* = 0.046). (C) Schematic representation of the *IFNG* promoter illustrating the location of analyzed CpG sites and the relative percent methylation change observed in participants with detectable HIV replication compared to participants with undetectable viral load. Significant CpG sites identified by adjusted beta-binomial analysis are indicated (*p* < 0.05).

Five CpG sites in the *IFNG* promoter were analyzed for differential DNA methylation across plasma viral load groups. Participants with high plasma HIV RNA exhibited lower methylation across four of five CpG sites compared to both medium and undetectable viral load groups. After adjustment for age and duration since infection using beta-binomial models, significant methylation differences persisted primarily between the medium and high viral load groups (Figure 1A, Table 2), with absolute methylation differences of approximately 9–11% across significant CpG sites. To further account for the potential confounding effects of ART exposure, we performed a sensitivity analysis restricted to participants not receiving ART. Partial Spearman correlation analysis adjusted for duration of infection demonstrated a significant inverse association between plasma HIV RNA and mean *IFNG* promoter methylation (*R* = −0.49, *p* = 0.046; Figure 1B), supporting an independent relationship between viral burden and promoter hypomethylation.

**Table 2.** Absolute percent methylation at each *IFNG* promoter CpG site across plasma HIV RNA groups.

CpG Site	Viral load group	% Methylation (Group)	% Methylation (High VL)	% Methylation change	*p*-value
−460	Undetectable	54.26	44.68	9.58	0.066
−460	Medium	55.38	44.68	10.7	0.005
−374	Undetectable	31.23	25.33	5.9	0.393
−374	Medium	31.06	25.33	5.73	0.165
−299	Undetectable	51.97	43.01	8.96	0.105
−299	Medium	52.9	43.01	9.89	0.011
−189	Undetectable	48.01	39.57	8.44	0.143
−189	Medium	48.22	39.57	8.65	0.030
−57	Undetectable	53.65	44.14	9.51	0.068
−57	Medium	54.79	44.14	10.65	0.005

Bars indicate the difference in observed mean methylation between the High viral load (VL) group and the Medium or Undetectable VL groups. *p*-values were calculated using beta-binomial models adjusted for age and duration since infection.

To compare the magnitude of these changes at each site, we determined the proportional increase in methylation in participants with undetectable and medium HIV RNA relative to those with high HIV RNA. All CpG sites examined in the medium HIV RNA group exhibited a consistent 6–10% absolute increase in methylation, with a proportional increase from 20–24% (Figure 1C), consistent with an association between viral antigen burden and *IFNG* promoter hypomethylation.

### High viral load correlates with elevated IFNG transcription

**Figure 2.**
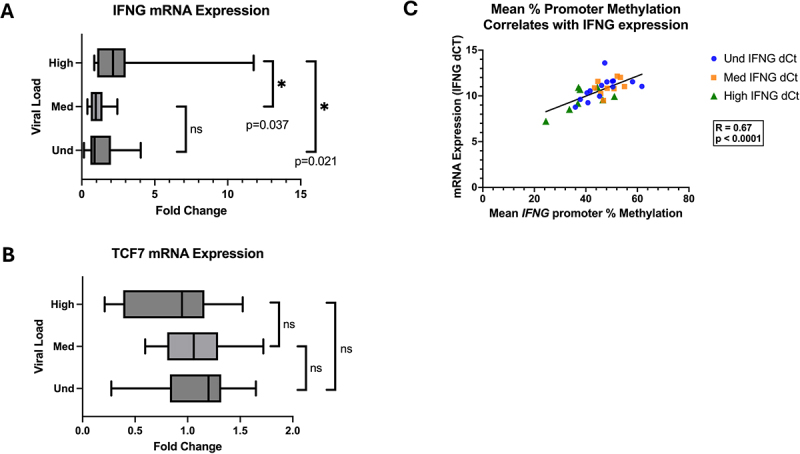
IFNG RNA expression correlates with HIV plasma RNA and *IFNG* promoter methylation. (A) Relative IFNG mRNA expression between viral load groups measured by quantitative RT-PCR. Expression values are shown as 2^-ΔΔCt normalized to RPP30. (B) Relative mRNA expression of T cell marker TCF7 across plasma viral load groups suggests a mild but non-significant reduction in T cells in PWH with high plasma HIV RNA. (C) IFNG mRNA dct (i.e., Higher IFNG dCt values correspond to lower IFNG mRNA expression) has a highly significant (*p* < 0.0001) positive correlation with average percent *IFNG* promoter CpG methylation. *p*-values for (A) and (B) were calculated using Kruskal-Wallis test for multiple comparisons followed by Dunn’s post-hoc analysis. Correlation analysis in (C) was calculated using Spearman rank correlation.

To identify whether the observed differential CpG methylation corresponds to transcriptional changes, we performed RT-qPCR for IFNG mRNA. While no significant difference in IFNG mRNA transcription was identified between the undetectable and medium plasma HIV RNA groups, PBMCs from high HIV plasma RNA participants expressed a significantly (~2-fold) higher IFNG mRNA compared to medium (*p* = 0.037) and undetectable (*p* = 0.021) HIV RNA participants (Figure 2A). To examine whether T cell marker expression differed significantly between viral load groups, we performed RT-qPCR for TCF7 mRNA, showing small, but not statistically significant differences between groups (Figure 2B). Additionally, to determine whether differential IFNG expression remained associated with plasma viral load independent of T cell abundance, we modeled IFNG expression while incorporating TCF7 expression as a covariate. Significant differences in IFNG expression between high viral load participants and both medium (*p* = 0.02) and undetectable viral load groups (*p* = 0.016) persisted after adjustment for TCF7 expression (Supplementary Table S3). Sensitivity analysis excluding the highest IFNG-expressing participant actually yielded similar directional associations with modestly stronger statistical significance in the TCF-7 adjusted model. These results show that IFNG transcription is significantly elevated in whole PBMCs of people with high HIV viral loads.

To examine the association between *IFNG* promoter CpG methylation and IFNG transcription, we performed Spearman’s rank correlation between mean *IFNG* promoter CpG methylation and IFNG RNA ΔCt, where a larger Ct denotes decreased RNA expression. Results showed a highly significant positive correlation between average promoter CpG methylation and IFNG RT-qPCR ΔCt (Spearman *R* = 0.67, *p* < 0.0001, Figure 2C), demonstrating increased *IFNG* promoter methylation is associated with decreased IFNG RNA expression. Additionally, mean *IFNG* promoter methylation was negatively correlated with *TCF7* ΔCt (Spearman *R* = −0.36, *p* = 0.047), consistent with higher TCF7 expression among samples with greater *IFNG* promoter methylation (Supplementary Figure S1). This supports the possibility that variation in T-cell abundance or T-cell differentiation state contributes to methylation patterns, but the viral load-associated methylation signal persisted after age/duration adjustment and ART-restricted sensitivity analyses.

To further evaluate whether the observed relationship between *IFNG* promoter methylation and IFNG expression could be explained by variation in T-cell abundance, we performed a partial correlation analysis adjusting for TCF7 expression. *IFNG* promoter methylation remained strongly associated with IFNG expression after adjustment for TCF7 (partial Spearman *R*=0.71, *p* < 0.0001; Supplementary Figure S2), indicating that the methylation-expression relationship was not solely attributable to differences in T-cell abundance or differentiation state.

## Discussion

Epigenetic immunoregulation represents a critical but understudied mechanism by which HIV shapes host antiviral responses. This study provides the first *in vivo* evidence that HIV RNA levels in plasma correlate with hypomethylation of the *IFNG* promoter and elevated IFNG transcription in PBMCs. Our results contrast with prior *in vitro* work, which reported HIV-induced hypermethylation at a single CpG site in the *IFNG* promoter, with a corresponding decrease in IFN-γ expression [33]. These contrasting results may reflect differences in study design, as the earlier work was conducted *in vitro* and used uninfected PBMCs as controls [33], while the main comparison group of this study was comprised of virally suppressed participants who still harbor HIV. Additionally, *in vitro* infection models are much shorter in duration, and typically yield a considerably higher percentage of infected cells (i.e., 10–30% within 2–3 days post-inoculation) than *in vivo* PBMC samples (typically <0.01% infected) [34–36].

Our findings reinforce broader evidence that HIV replication influences epigenetic regulation of interferon pathways. Lymphocytes from viremic individuals have been shown to exhibit hypomethylation across genes involved in IFN signaling [12]. Similarly, we documented decreased *IFNG* promoter methylation concomitant with increasing HIV RNA, further paralleled by increased IFNG mRNA expression. These findings support viral burden-associated epigenetic remodeling of host immune genes in circulating immune cells, consistent with increased IFN-γ responses during higher viremic states [12]. These results also align with an early study showing that CpG demethylation of the *IFNG* promoter correlated with increased IFNG transcription [37], as well as several developmental studies reporting differential DNA methylation in CD4+ T cell subsets [15–17].

Immunological contexts have been suggested to underlie biological drivers of differential methylation outcomes, and ultimately immune response mechanisms. DNA demethylation at the *IFNG* locus has been shown to be dependent on TET2, mediated by IL-12 signaling [38]. Furthermore, in T cells from mice, Tet2 has been shown to colocalize with regulatory DNA elements and transcription factors, and demethylation at these sites [39]. The deletion of Tet2 in mice resulted in inhibited cytokine expression, resulting in the reduced colocalization of these factors and elevated methylation rates [39]. These mechanisms suggest that increased inflammatory signaling during HIV infection may be associated with TET2 recruitment and mediated demethylation at the *IFNG* promoter, altering host immune responses.

Together, our evidence suggests that *IFNG* promoter demethylation emerges preferentially during higher viremic states, rather than as a gradual response to increasing viral load. Such threshold-dependent demethylation is consistent with established epigenetic models in which DNA methylation functions to stabilize transcriptional states that are initiated by earlier gene activation events [40–42]. The lack of significant methylation differences between the undetectable and medium viral load groups may reflect persistent epigenetic remodeling in the face of viral suppression. This possibility is consistent with the concept of epigenetic ‘memory,’ in which prior inflammatory or viremic states may leave durable methylation signatures even after ART-mediated suppression.

Limitations of this study include the cross-sectional design, PBMC cell type heterogeneity, small sample size, and ART imbalance across groups. The cross-sectional design of the study precludes any causal inference, and PBMC heterogeneity also limits resolution of cell-specific effects without cell type deconvolution. Furthermore, ART imbalance introduces a potential confounder, as all participants with undetectable HIV RNA were on ART, in contrast to those with medium and high HIV RNA. Because these analyses were performed using bulk PBMCs, future studies using sorted immune subsets and longitudinal sampling will be required to establish whether *IFNG* promoter methylation persists longitudinally after ART suppression.

Our study was intentionally designed as a focused *in vivo* epigenetic association analysis rather than a mechanistic dissection of chromatin remodeling pathways. However, our results illustrate a potential mechanism by which HIV impacts host chromatin modifications to shape immune responses. Future investigation of the longitudinal progression of chromatin accessibility at these sites will help decipher how host epigenetic machinery affects interferon pathways, potentially enabling viral persistence.

## Conclusion

This study identifies a robust association between plasma HIV RNA and CpG-specific methylation of the *IFNG* promoter, accompanied by corresponding differences in IFNG transcription. These findings support epigenetic modulation of a key immune regulatory locus in the context of ongoing HIV antigen exposure after adjustment for age and duration of infection, with additional sensitivity analyses supporting that these associations were not solely attributable to ART exposure. Our results illustrate how focused, locus-resolved epigenetic analyses can reveal transcriptionally meaningful host responses in chronic viral infection. These findings provide a focused *in* *vivo* framework for understanding locus-specific epigenetic responses to viral burden.

## Supplementary Material

SupplementaryTable3_20260528.png

SupplementaryTable1_Revised_20260126.png

Supp_Fig_1.png

SupplementaryTable2_20260528.png

Supplementary_Figure_2.png

## Data Availability

Sequencing data generated in this study has been deposited in the NCBI Sequence Read Archive (Bioproject PRJNA1472803).
