# Supplementary figures and images for: Differential CpG methylation in the Interferon Gamma (IFNG) promoter correlates with viral load and transcriptional control in people with HIV
Source: Epigenetics. 2026 Jun 30;21(1):2693337. doi: 10.1080/15592294.2026.2693337 (PMC13327351; doi:10.1080/15592294.2026.2693337)

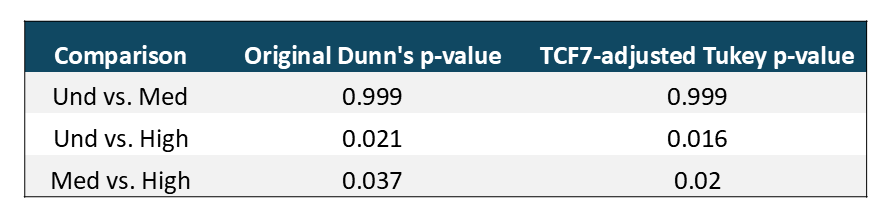

Supplement: SupplementaryTable3_20260528.png [file KEPI_A_2693337_SM9207.png]

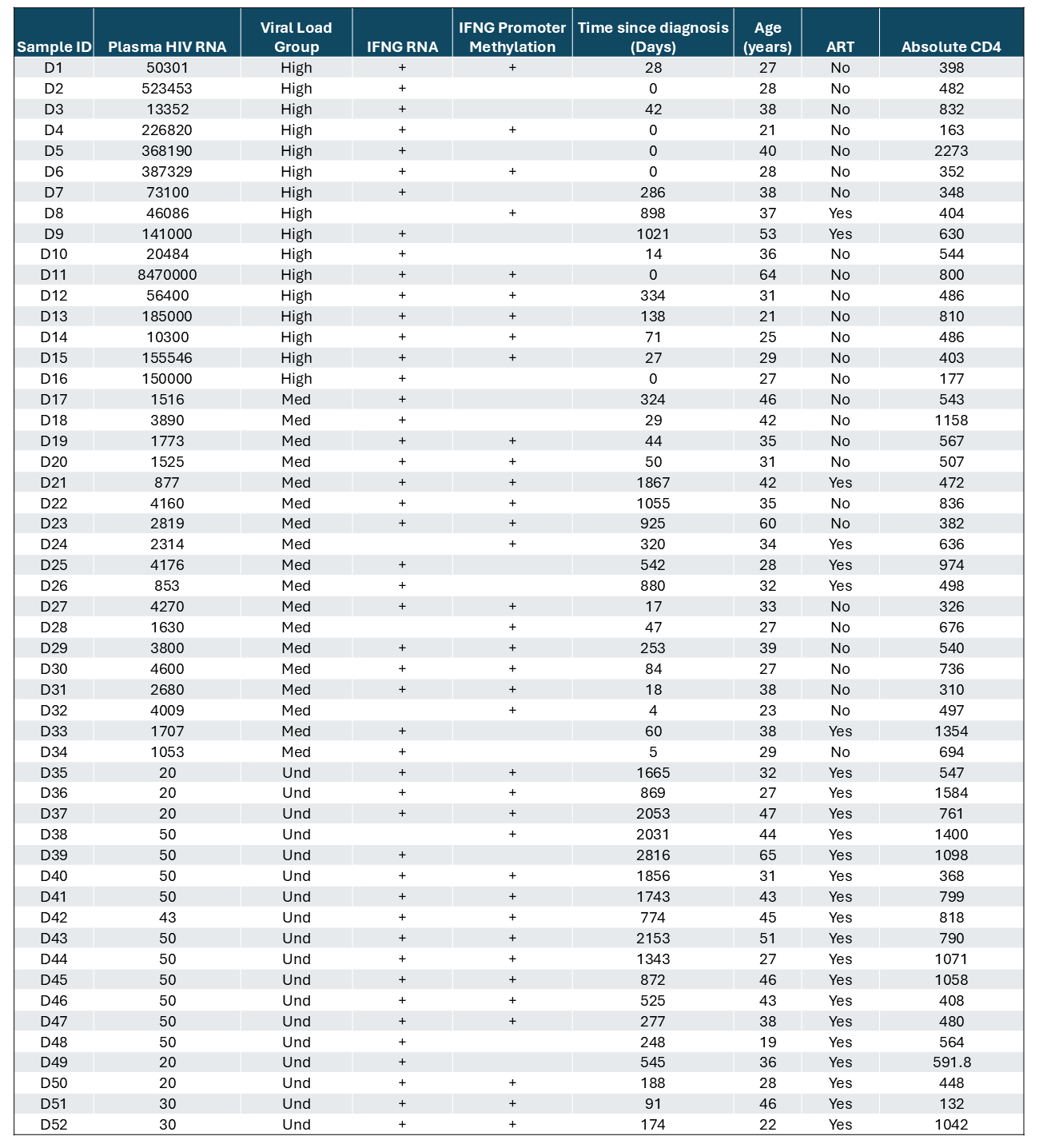

Supplement: SupplementaryTable1_Revised_20260126.png [file KEPI_A_2693337_SM9206.png]

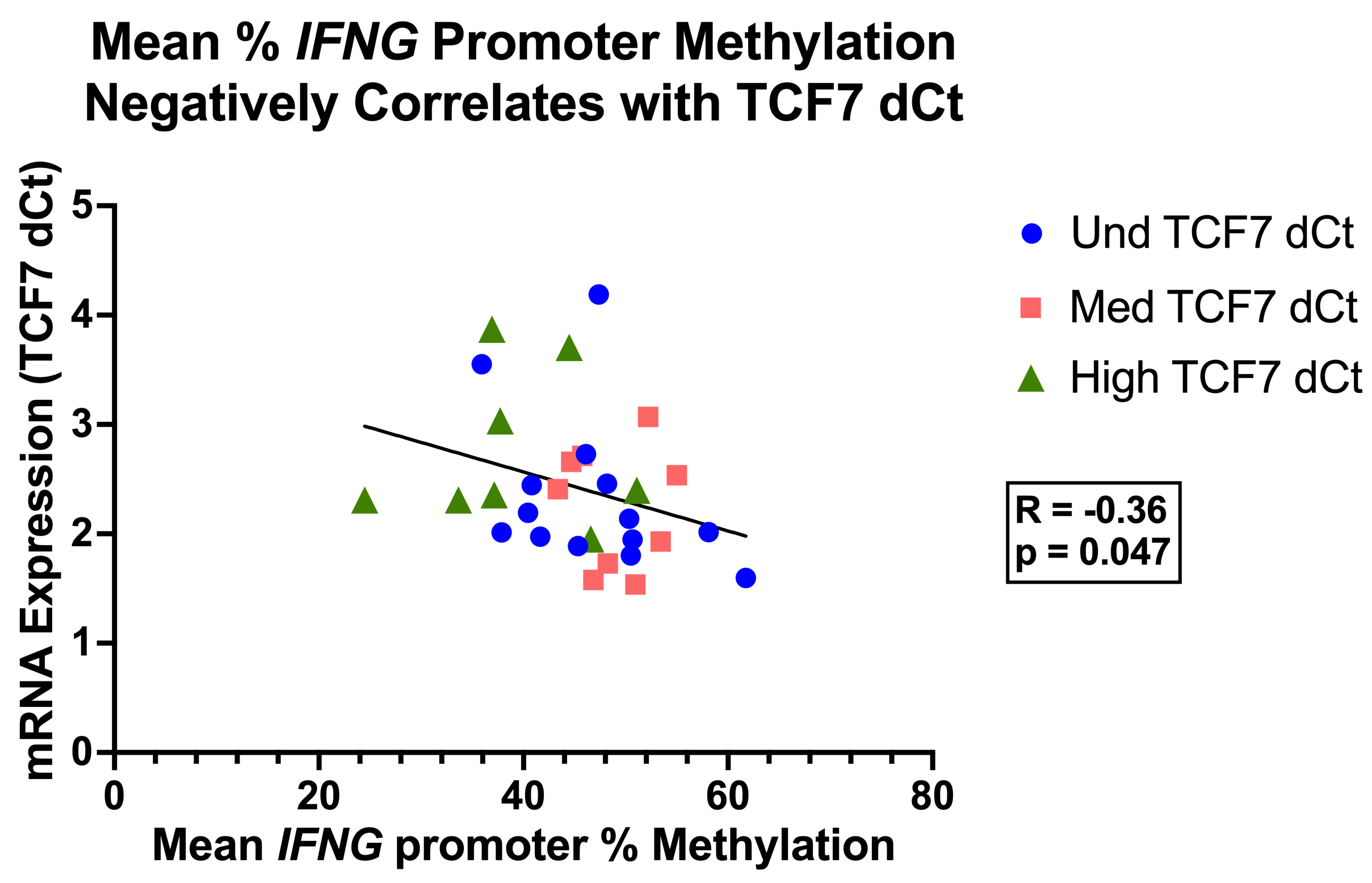

Supplement: Supp_Fig_1.png [file KEPI_A_2693337_SM9205.png]

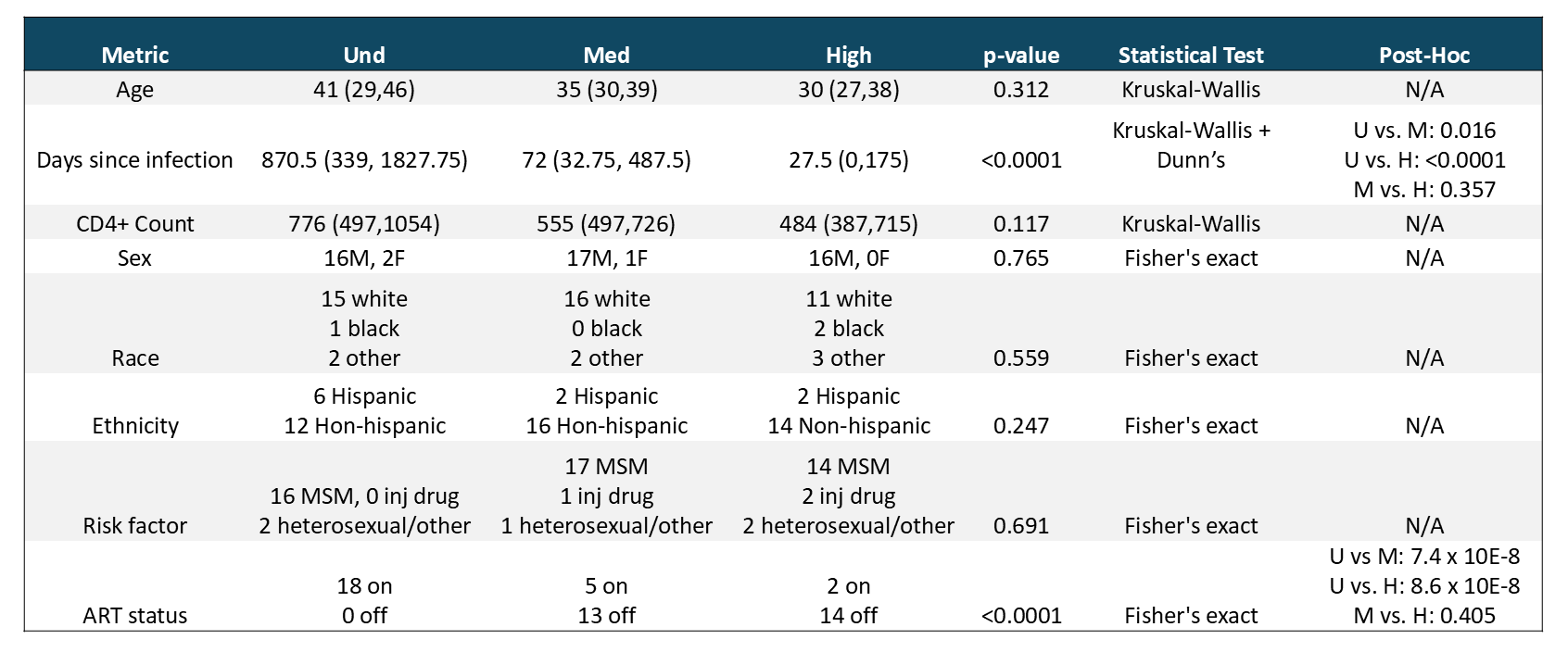

Supplement: SupplementaryTable2_20260528.png [file KEPI_A_2693337_SM9204.png]

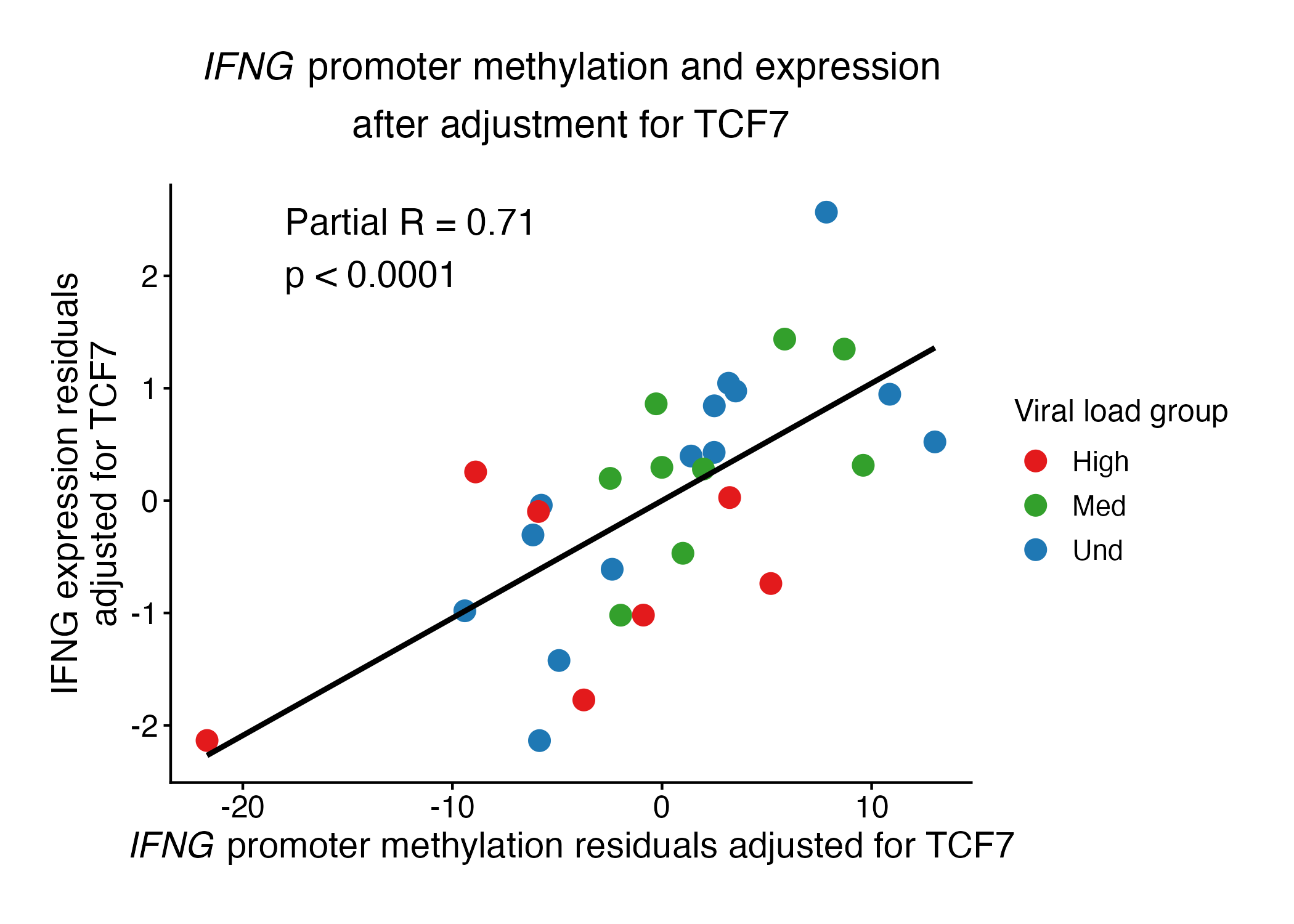

Supplement: Supplementary_Figure_2.png [file KEPI_A_2693337_SM9203.png]
